# Pilot Trial of FANG Immunotherapy in Ewing's Sarcoma

**DOI:** 10.1038/mt.2015.43

**Published:** 2015-04-28

**Authors:** Maurizio Ghisoli, Minal Barve, Reva Schneider, Robert Mennel, Carl Lenarsky, Gladice Wallraven, Beena O Pappen, John LaNoue, Padmasini Kumar, Derek Nemunaitis, Alyssa Roth, James Nemunaitis, Sam Whiting, Neil Senzer, Frederick A Fletcher, John Nemunaitis

**Affiliations:** 1 Mary Crowley Cancer Research Centers, Dallas, Texas, USA; 2 Texas Oncology, P.A., Dallas, Texas, USA; 3 Gradalis, Inc., Dallas, Texas, USA; 4 Medical City Dallas Hospital, Dallas, Texas, USA; 5 Baylor University Medical Center, Dallas, Texas, USA; 6 Medical City Children's Hospital, Dallas, Texas, USA

## Abstract

We report on 12 consecutive patients with advanced/metastatic Ewing's sarcoma who were treated as a separate cohort of a phase 1 trial of FANG autologous immunotherapy (1 × 10^6^–2.5 × 10^7^ cells/intradermal injection each month for minimum 4 months). Safety and clinical response were monitored. Patient immune response to unmodified autologous tumor cells was assessed by gamma interferon-enzyme-linked immunospot (γIFN-ELISPOT) assay using peripheral blood mononuclear cells from baseline (pretreatment) and multiple postvaccination time points. None of the 12 patients (47 vaccinations) developed grade 2/3/4 drug-related toxicity. Median product release granulocyte-macrophage colony-stimulating factor expression was 1,941 pg/10^6^ cells, and TGFβ1and TGFβ2 knockdown were 99 and 100%, respectively. Eight patients were assessed for ELISPOT response to autologous tumor cells at baseline and all (100%) were negative. In contrast, follow-up ELISPOT response at month 1 or month 4 (one patient) after FANG was positive in all eight patients. One patient achieved a partial tumor response (38% tumor reduction, RECIST 1.1). The Kaplan–Meier estimated survival of these 12 patients at 1 year was 75%. In this phase 1 study in patients with Ewing's sarcoma, FANG immunotherapy was well tolerated, elicited a tumor-specific systemic immune response in all patients, and was associated with favorable 1-year survival. Further clinical testing is indicated.

## Introduction

Ewing's sarcoma (EWS) is a rare adolescent malignant bone tumor distinguished by a translocation of the EWS gene on chromosome 22q12 with one of the E26 transformation-specific transcription factory family genes.^1^ Up to 85% of Ewing's tumors are characterized by the (11;22)(q24;12) translocation resulting in the EWS/FLI1 fusion gene.^2^ The median age of diagnosis for adolescents with EWS is 14 years.^3,4^ The 5-year survival with standard of care is ~30% for EWS patients with metastatic lesions isolated to the lung and <20% for those with bone or bone marrow involvement.^3,5,6^ In patients refractory, resistant, or otherwise failing first-line therapy, survival at 5 years is even more severely limited,^4,7,8,9,10,11,12^ particularly in those who relapse within 2 years of frontline treatment. In one large retrospective analysis of 714 patients from the time of first relapse, 5-year overall survival (OS) was 13%.^4^ In another study focusing on relapses that occur within the first 2 years after initial diagnosis, which make up 72% of relapses,^4^ the 2-year OS was 7%.^9^ The 5-year survival, following failure to respond to second-line treatment, is only 4%.^13^ Moreover, the toxicity profile of standard (year-long) frontline chemotherapy was characterized by significant morbidity and rare mortality.^14^

The potential for efficacy of an immunotherapeutic approach is suggested by the finding that EWS tumor samples taken at the time of initial diagnosis, which exhibit higher numbers of tumor-infiltrating CD8+ T-lymphocytes, correlate with lower tumor volume and better OS (*P* = 0.05).^15^ In another evaluation, mice immunized with tumor peptides having modified anchor residues generated cytotoxic T-cells, which were active against human EWS cell lines.^16^ These cytotoxic CD8 T-cells increased survival when transferred to severe combined immunodeficiency mice previously inoculated with human EWS cells. However, the investigators noted that native peptides showed weak affinity to HLA-A2.1 with poor stability of peptide/ major histocompatibility complex (MHC) complexes. Further, 79% of Ewing's tumors showed almost complete absence of human leukocyte antigen (HLA) class I expression, as well as a lack of functional class II transactivator manifesting as impaired HLA class II expression.^17^

In a previous publication, we established the safety of FANG immunotherapy and showed a correlation of induced T-cell activation (gamma interferon-enzyme-linked immunospot (γIFN-ELISPOT)) with survival in adults with multiple cancer types.^18,19,20^ The FANG immunotherapy comprises autologous tumor cells as a source of the tumor-specific antigenic matrix transfected with the rhGMCSF transgene and the RNAi bi-shRNA^furin^ to establish a “triad” functionality—(i) patient tumor-specific antigen presentation, (ii) dendritic cell (DC) recruitment, activation and enhanced regional nodal migration (granulocyte-macrophage colony-stimulating factor (GMCSF)), and (iii) reversion of immune tolerance (by blocking furin activation of endogenous TGFβ1 and TGFβ2).^18,19,20^ We now report a pilot experience of FANG immunotherapy in advanced EWS patients with recurrent or refractory disease.

## Results

### Patient demographics

Twenty-seven consecutive tumor specimens were harvested from 25 consecutive EWS patients (two patients underwent a second additional harvest, #s 2, 5), and 175 vaccine vials were successfully manufactured. Four patient samples had insufficient tumor cells harvested, and seven patient samples failed release criteria due to bacterial contaminant (introduced during surgical harvest prior to immunotherapy construction). Two patients had successful manufacture of therapy but elected to not move forward with the FANG treatment. Twelve consecutive EWS patients thus were treated (demographics in **Table 1**). All 12 patients had metastatic disease and were either multiply recurrent (*n* = 11) or had failed frontline treatment within 2 years (*n* = 1). One patient (#2) received two FANG immunotherapy treatments from two separate tissue procurements.

### Construction/release

All of the vaccines of the 12 consecutive patients treated fulfilled the QA release criteria including adequate GMCSF production (median: 1,941 pg, range: 31–14,751 pg) and TGFβ1 (median: 99%, range: 84–100%) and TGFβ2 (median: 100%, range: 84–100%) knockdown (**Supplementary Table S1**).

### Response/safety

**Figure 1:**
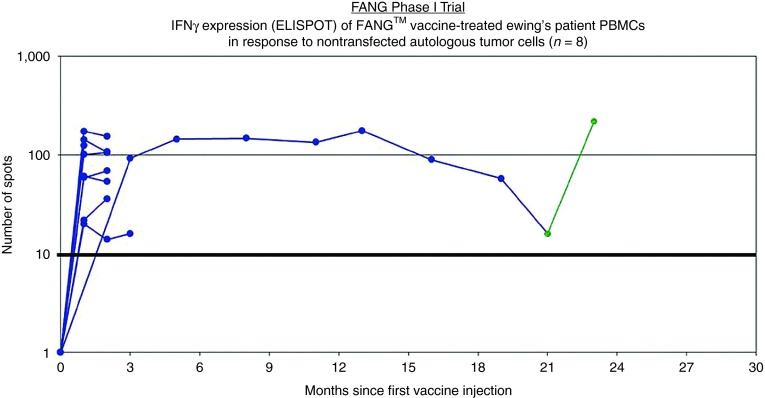
**Gamma interferon (γIFN) expression (enzyme-linked immunospot (ELISPOT)) of FANG vaccine-treated Ewing's sarcoma (EWS) patient PBMCs over time in response to nontransfected autologous tumor cells (*n* = 8).** One patient had a second vaccine constructed with solitary lesion progression (patient #2, sample 098). Positive ELISPOT activation was again developed to the second harvested autologous tumor sample (green) (data as of 10/13/14). PBMC, peripheral blood mononuclear cell.

**Figure 2:**
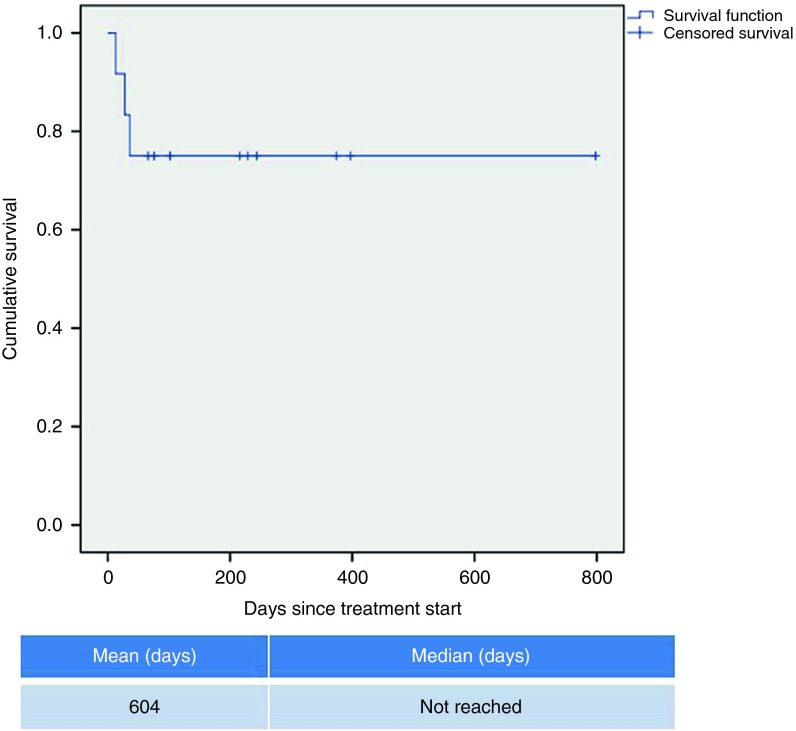
**Kaplan–Meier survival curve of treated EWS patients (*n* = 12).**

All patients received at least one vaccination. No grade 2, 3, and 4 toxic effects related to FANG were observed. Side effects were limited to grade 1 primary local reactions (erythema, induration, bruise, and pain). Eight patients were evaluated for circulating immune response to unmodified autologous tumor by the IFNγ-ELISPOT assay. As shown in **Figure 1**, all eight patients were negative by IFNγ-ELISPOT assay at baseline and all eight converted to a positive ELISPOT response at month 1 (*n* = 7) or month 4 (*n* = 1, patient #2 was not measured at month 1). Patient outcomes are summarized in **Table 2** and patient survival estimated by the Kaplan–Meier method is shown in **Figure 2**.

Two cases warrant further discussion. The first case (patient #2 in **Table 1**) is a patient who had a second *de novo* FANG constructed from tumor cells obtained from the single solitary site of progression in her lung (*i.e.*, first vaccine was 062 and the second vaccine was 098). The patient continues disease-free at >2 years post-procurement, which is of longer duration than her first disease-free interval.

**Figure 3:**
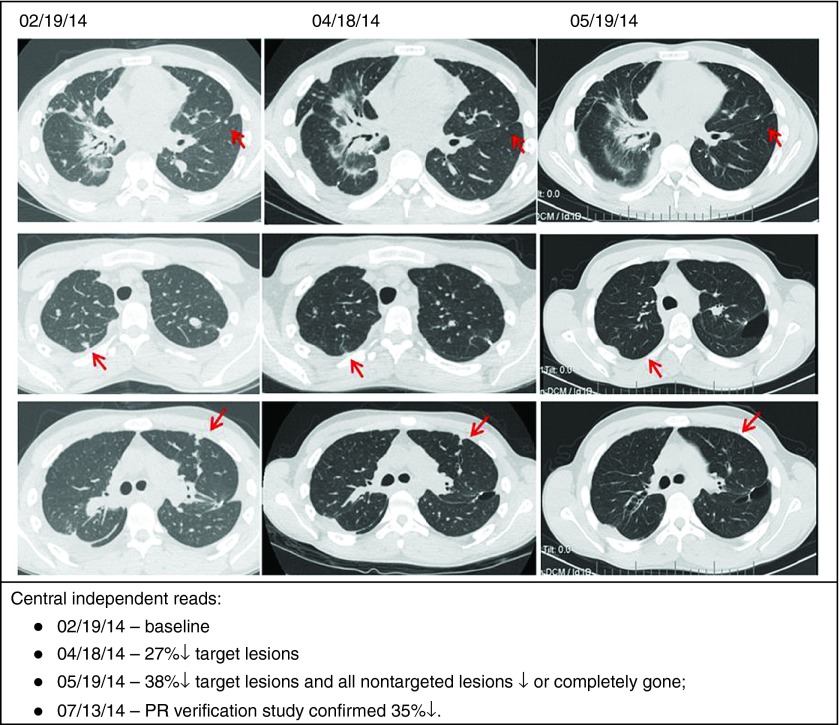
**Status of patient #6 in Table 1: post frontline HD chemotherapy, vincristine/irinotcan/temodar, cixutumumab/timsirolimus, pazopanub/everolimus, ifosfamide/etoposide, meckinist/rapamycin/metformin, HD ifosfamide→surgery→FANG ×4.**

A second case (*i.e.*, patient #6 in **Table 1**) is that of a patient with advanced disease who achieved an objective partial response following FANG vaccine (**Figure 3**). This patient also had a positive ELISPOT response from 0 spots at baseline to 174 at month 1 and 155 at month 2.

## Discussion

Few EWS patients respond to second-line therapy and there is no standard of care second-line treatment. Regimens such as topotecan/cyclophosphamide, irinotecan/temozolomide, or docetaxel/gemcitabine are second-line treatment options with less than 15% response rate and limited evidence for prolongation of life despite modest toxicity.^4,7,8,9,10,11,12^ An even worse outcome is predicted for patients refractory to frontline or second-line treatment.^13^ An alternative, perhaps complementary, therapeutic strategy to breach the second-line impasse is the targeted application of the recent advances in molecular immunology and technologies that have already been translated into positive clinical results. Although historically the sarcomas as a whole have shown disappointing clinical immunoresponsiveness, recent research and clinical findings have led to a renewed enthusiasm. Preclinical studies have shown the effectiveness of cytotoxic CD8 T-cell targeting of the EWS/FLI1 fusion gene-specific expressed antigens, including EZH2 and CHM1 (ref. 23) and an array of differentially expressed cancer testes antigens.^24^ The number of tumor-infiltrating CD8+ T-lymphocytes correlates with better OS (*P* = 0.05)^15^ as well as with the expression levels of HLA class 1,^25^ which however are absent in a majority of EWS tumors.^17^ Therefore, the potential importance of the finding that the shRNA-mediated downregulation of APLP2 (the expression of which is further enhanced by radiation) results in an increase in MHC class I expression.^26^ Further, the ganglioside G_D2_ that has been shown to be a targetable antigen is a carbohydrate not requiring MHC class I presentation.^27,28^ Additionally, the therapeutic potential of natural killer cell-mediated cytotoxicity has begun to be explored in EWS tumors.^29^ Finally, with regard to successful clinical translation of these possibilities, a recent trial of consolidative immunotherapy in pediatric sarcomas including high-risk EWS not only showed provocative 5-year OS but also suggested the persistence of intact immune pathways in the postchemotherapy population.^30^

Control of TGFβ1 and TGFβ2 is a unique aspect of the FANG technology. Transforming growth factors beta (TGFβ) are a family of multifunctional proteins that regulate the growth and function of many normal and neoplastic cell types.^31,32,33,34^ Proteolytic cleavage by the proprotein convertase furin is required for TGFβ activation (*i.e.*, pro-TGFβ→TGFβ). The dimeric TGFβ activates a tetrameric TGFβ receptor complex comprised of TGFβRII and TGFβRI (ALK5) resulting in the phosphorylation of Smad2 and Smad 3, which translocate to the nucleus complexed with Smad4 where a number of transcription factors are engaged. TGFβ exerts a wide range of effects on a variety of cell types and has been shown to stimulate or inhibit cell growth, induce apoptosis and increase angiogenesis.^35,36,37,38,39^ Although TGFβ has been shown to be an effective tumor suppressor in epithelial cells in the early phases of tumorigenesis, once the tumor escapes its growth regulatory effects, likely as the result of genetic instability, TGFβ appears to function as a tumor promoter^40,41^ by virtue of its involvement in all six of the essential hallmark cancer-related processes as defined by Hanahan and Weinberg.^42^ Overexpression of TGFβ(s) correlates with tumor progression and poor prognosis^40,43^ in many types of cancer, including soft tissue sarcomas in which one analysis of 249 patients showed that elevated tumor expression of TGFβ significantly correlated with poor disease-specific survival.^44^ Thus, control of TGFβ(s) expression could potentially be used as a justification for anticancer immune induction.

Furthermore, elevated TGFβ2 levels are linked with immunosuppression in both the afferent and efferent limbs of the immune response arc,^31,32,33,40,45–47^ although there is some evidence to suggest that TGFβ predominantly affects the afferent limb of the immune response and that it does not suppress the function of activated effector cells.^48^ Tumor-derived TGFβ1 and PGE2 induce the upregulation of PD-L1 in immunocompetent splenic DCs and are causally related to the shift in DC phenotype from immunostimulatory to immunosuppressive in the transgenic LSL-K-rasG12D/+p53loxP/loxP murine model of induced metastatic ovarian cancer.^49^ TGFβ2 inhibits T-cell activation in response to antigen stimulation as well as targeting cytotoxic T-cell cytolytic pathways.^50^ Additionally, TGFβ2 has antagonistic effects on the natural killer cells as well as the induction and proliferation of lymphokine-activated killer cells.^51,52,53,54,55,56^

The immune suppressor functions of TGFβ proteins thus are well characterized and accepted and are likely to play a major role in modulating the effectiveness of cancer-cell vaccines. TGFβ inhibits GMCSF-induced maturation of bone marrow-derived DCs^57^ as well as expression of MHC Class II and co-stimulatory molecules.^58^ It has been shown that antigen presentation by immature DCs result in T-cell unresponsiveness.^59^ TGFβ also inhibits activated macrophages^60^ including their antigen-presenting function.^61,62^ Both the immunosuppressive effects of elevated TGFβ isoforms in malignant cells, including the inhibitory effects of these isoforms on GMCSF immune modulatory function, support a broad-based tumor target range for the application of a TGFβ-suppressed/GMCSF-expressing immune enhancing therapeutic. The triad FANG vaccine provides a immune-enhancing therapeutic activity by enhancing (i) patient tumor-specific antigen presentation, (ii) DC recruitment, activation and regional nodal migration (GMCSF), and by (iii) reversion of immune tolerance (by blocking furin activation of endogenous TGFβ1 andTGFβ2).

Our previously published phase 1 FANG trial, which involved adults, established product safety and confirmed GMCSF transgene expression and effective silencing of furin expression and consequent knockdown of TGFβ1 and TGFβ2 expressions. The study showed a 54% conversion from ELISPOT negative status at baseline to ELISPOT positive status postvaccination using the patient's autologous tumor cells as the antigen source. It is provocative that the study also showed a correlation between a FANG-elicited conversion to positive ELISPOT response and OS.^19,20^ A separate ongoing randomized, controlled, phase 2 trial of FANG in maintenance treatment of frontline ovarian cancer patients suggests a time to recurrence advantage over control and further supports a clinical benefit related to the “triad” vaccine concept.^63,64^

Here we report the immune response and preliminary survival data of an expansion cohort of the FANG phase 1 study focusing upon patients with refractory EWS. All of these patients were heavily pretreated of high risk with metastatic disease. They either had early relapse following first line therapy or had multiple recurrent or chemotherapy refractory disease. It is notable that in this population of EWS patients 100% were IFNγ-ELISPOT negative at baseline and 100% converted to IFNγ-ELISPOT positive following FANG treatment. This immune conversion rate compares very favorably to the 54% rate seen in the phase 1 trial as a whole. It is possible that the young age of the EWS patients contributes to this dramatic immune response. However, an intriguing hypothesis is that the presence of a nonself, mutated, neoantigen (the EWS fusion protein) in nearly all EWS patients results in the presence of high-affinity T cells not subjected to prior central tolerance. The FANG treatment may facilitate the activation of those T cells via DC cross presentation much better than unmodified EWS cells—particularly given the report of MHC class I down regulation on the EWS cells. Also provocative in these EWS patients is the Kaplan–Meier estimated 1-year survival of 75%. While unproven, it is intriguing to consider that a causal relationship may exist between the high induction of anti-tumor cellular immune response induced by FANG in these EWS patients and preliminary evidence of favorable 1-year survival.

A phase 2 randomized study comparing FANG to second-line chemotherapy in pediatric patients with EWS is in preparation.

## Materials and Methods

The construction and current good manufacturing practice manufacturing of FANG immunotherapy have previously been described.^19,21^ Briefly, the FANG vector utilizes the pUMVC3 vector backbone in which the GMCSF encoding complementary DNA and the DNA encoding the furin bifunctional shRNA are under transcriptional control of the cytomegalovirus immediate-early promoter. The final construct was confirmed by bi-directional sequencing.

Following protocol-specific informed consent, the tumor was excised, placed in sterile transport media, and brought to the Gradalis manufacturing facility (Carrollton, TX).

The FANG immunotherapy is manufactured over two consecutive days by first dissociating the tumor cells into a single-cell suspension, then electroporating the FANG plasmid into the cells, followed by overnight incubation. The next day cells are irradiated (100 Gray), cryopreserved and good manufacturing practice Quality Assurance (QA) release testing initiated. Only after successful completion of QA release testing can patients be treated.

### Study design

The primary objective of this phase 1, non-randomized, open label trial (previously described in (ref. 19) was to evaluate the safety of the FANG immunotherapy in patients with advanced solid tumors who did not have an alternative standard therapy or curative options. Following progression on previous therapy, the patients were entered into the study depending on the manufacturing cell yield from the harvested tumor, using a minimum criteria of four monthly injections at either 1 × 10^6^ cells/injection, 4.0 × 10^6^ cells/injection, 8.3 × 10^6^ cells/injection, 1 × 10^7^ cells/injection or 2.5 × 10^7^ cells/injection. The vaccine, in a 1-ml injection volume, was administered monthly to a maximum of 12 intradermal injections alternating between the right and left upper arms. The approval for an amendment to the ongoing phase 1 trial was obtained to justify treatment of the extension cohort of EWS patients described in this manuscript. The details of methods including radiographic image, lab assessment and tumor response criteria have been published.^19^

Eligibility requirements included the manufacture of a minimum of four immunotherapy doses. Treatment was continued until documentation of progressive disease or to a maximum of 12 injections.

The trial was performed after approval by a local Ethics Committee and in accordance with an assurance filed with and approved by the Department of Health and Human Services.

### Patient population

All eligible patients were treated in the outpatient facilities of Mary Crowley Cancer Research Centers (MCCRC), Dallas, TX. Specific inclusion criteria have been previously described.^19^

### Enzyme-linked immunospot (ELISPOT) assay

Gamma interferon-enzyme-linked immunospot (γIFN-ELISPOT) assay was performed as previously described using ELISPOT for γIFN (BD Biosciences, San Jose, CA) and the patient's unmodified whole tumor cells as antigen source.^19,22^ Independent reading of ELISPOT plates was performed by ZellNet Consulting (Fort Lee, NJ). A value of ≥10 spots and >2× baseline was considered a positive response. The ELISPOT analyses were performed on patients at baseline and sequentially starting at month 1 postinitiation of vaccination.

**SUPPLEMENTARY MATERIAL** **Table S1.** Release criteria of vaccines constructed for treated patients

## Figures and Tables

**Table 1:**
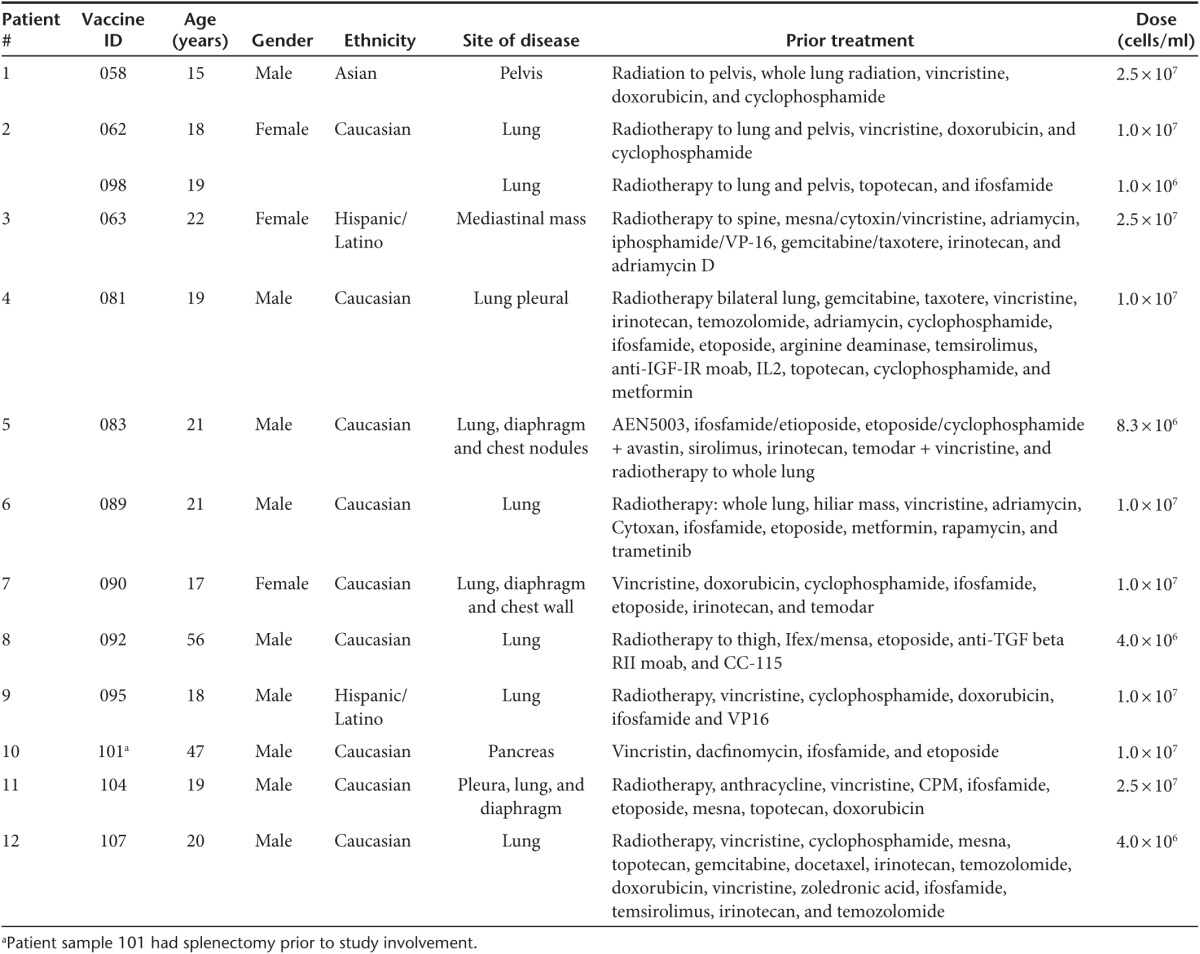
Demographics of FANG-treated Ewing's sarcoma patients (data as of 13 October 2014)

**Table 2:**
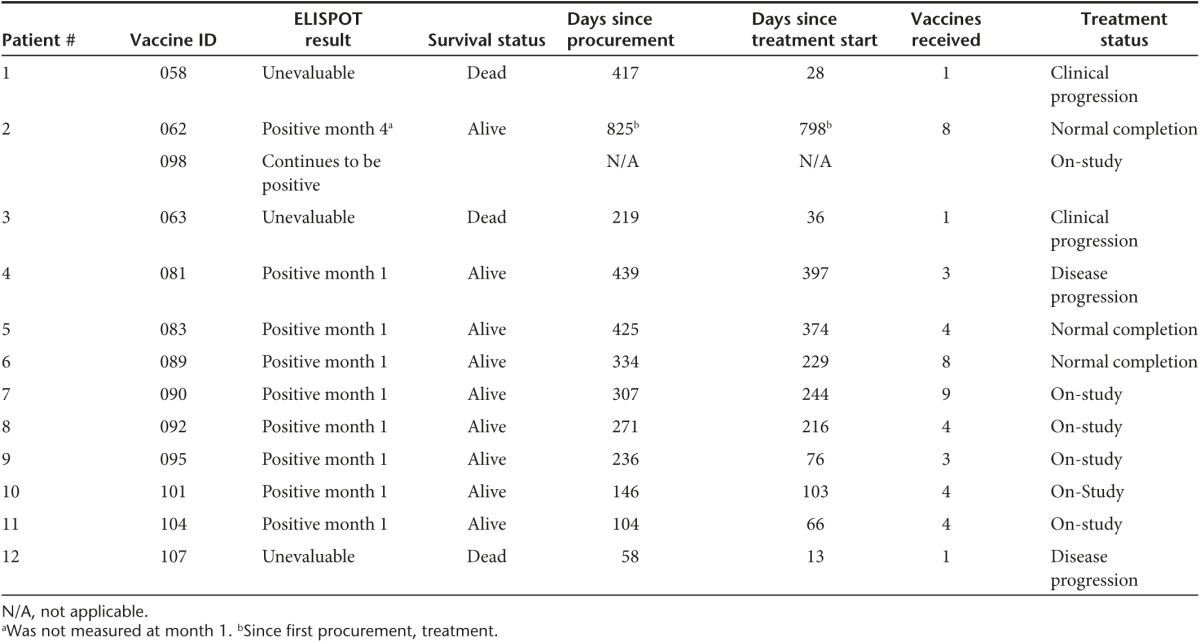
Response of FANG treated Ewing's sarcoma patients

## Supplementary Material

Supplementary Table S1 Release criteria of vaccines constructed for treated patients

## References

[bib1] Mariño-Enríquez, A and Fletcher, CD (2014). Round cell sarcomas - biologically important refinements in subclassification. Int J Biochem Cell Biol 53: 493–504. 24801613 10.1016/j.biocel.2014.04.022

[bib2] Arvand, A and Denny, CT (2001). Biology of EWS/ETS fusions in Ewing's family tumors. Oncogene 20: 5747–5754. 11607824 10.1038/sj.onc.1204598

[bib3] Cotterill, SJ, Ahrens, S, Paulussen, M, Jürgens, HF, Voûte, PA, Gadner, H et al. (2000). Prognostic factors in Ewing's tumor of bone: analysis of 975 patients from the European Intergroup Cooperative Ewing's Sarcoma Study Group. J Clin Oncol 18: 3108–3114. 10963639 10.1200/JCO.2000.18.17.3108

[bib4] Stahl, M, Ranft, A, Paulussen, M, Bölling, T, Vieth, V, Bielack, S et al. (2011). Risk of recurrence and survival after relapse in patients with Ewing sarcoma. Pediatr Blood Cancer 57: 549–553. 21442722 10.1002/pbc.23040

[bib5] Meyers, PA, Krailo, MD, Ladanyi, M, Chan, KW, Sailer, SL, Dickman, PS et al. (2001). High-dose melphalan, etoposide, total-body irradiation, and autologous stem-cell reconstitution as consolidation therapy for high-risk Ewing's sarcoma does not improve prognosis. J Clin Oncol 19: 2812–2820. 11387352 10.1200/JCO.2001.19.11.2812

[bib6] Grier, HE, Krailo, MD, Tarbell, NJ, Link, MP, Fryer, CJ, Pritchard, DJ et al. (2003). Addition of ifosfamide and etoposide to standard chemotherapy for Ewing's sarcoma and primitive neuroectodermal tumor of bone. N Engl J Med 348: 694–701. 12594313 10.1056/NEJMoa020890

[bib7] Rodriguez-Galindo, C, Billups, CA, Kun, LE, Rao, BN, Pratt, CB, Merchant, TE et al. (2002). Survival after recurrence of Ewing tumors: the St Jude Children's Research Hospital experience, 1979-1999. Cancer 94: 561–569. 11900241 10.1002/cncr.10192

[bib8] Bacci, G, Ferrari, S, Mercuri, M, Longhi, A, Giacomini, S, Forni, C et al. (2003). Multimodal therapy for the treatment of nonmetastatic Ewing sarcoma of pelvis. J Pediatr Hematol Oncol 25: 118–124. 12571462 10.1097/00043426-200302000-00007

[bib9] Shankar, AG, Ashley, S, Craft, AW and Pinkerton, CR (2003). Outcome after relapse in an unselected cohort of children and adolescents with Ewing sarcoma. Med Pediatr Oncol 40: 141–147. 12518341 10.1002/mpo.10248

[bib10] Barker, LM, Pendergrass, TW, Sanders, JE and Hawkins, DS (2005). Survival after recurrence of Ewing's sarcoma family of tumors. J Clin Oncol 23: 4354–4362. 15781881 10.1200/JCO.2005.05.105

[bib11] McTiernan, AM, Cassoni, AM, Driver, D, Michelagnoli, MP, Kilby, AM and Whelan, JS (2006). Improving Outcomes After Relapse in Ewing's Sarcoma: Analysis of 114 Patients From a Single Institution. Sarcoma 2006: 83548. 17496997 10.1155/SRCM/2006/83548 PMC1698143

[bib12] Leavey, PJ and Collier, AB (2008). Ewing sarcoma: prognostic criteria, outcomes and future treatment. Expert Rev Anticancer Ther 8: 617–624. 18402528 10.1586/14737140.8.4.617

[bib13] Rasper, M, Jabar, S, Ranft, A, Jürgens, H, Amler, S and Dirksen, U (2014). The value of high-dose chemotherapy in patients with first relapsed Ewing sarcoma. Pediatr Blood Cancer 61: 1382–1386. 24729428 10.1002/pbc.25042

[bib14] Esiashvili, N, Goodman, M and Marcus, RB Jr (2008). Changes in incidence and survival of Ewing sarcoma patients over the past 3 decades: Surveillance Epidemiology and End Results data. J Pediatr Hematol Oncol 30: 425–430. 18525458 10.1097/MPH.0b013e31816e22f3

[bib15] Berghuis, D, Santos, SJ, Baelde, HJ, Taminiau, AH, Egeler, RM, Schilham, MW et al. (2011). Pro-inflammatory chemokine-chemokine receptor interactions within the Ewing sarcoma microenvironment determine CD8(+) T-lymphocyte infiltration and affect tumour progression. J Pathol 223: 347–357. 21171080 10.1002/path.2819

[bib16] Evans, CH, Liu, F, Porter, RM, O'Sullivan, RP, Merghoub, T, Lunsford, EP et al. (2012). EWS-FLI-1-targeted cytotoxic T-cell killing of multiple tumor types belonging to the Ewing sarcoma family of tumors. Clin Cancer Res 18: 5341–5351. 22879388 10.1158/1078-0432.CCR-12-1985 PMC3463738

[bib17] Berghuis, D, de Hooge, AS, Santos, SJ, Horst, D, Wiertz, EJ, van Eggermond, MC et al. (2009). Reduced human leukocyte antigen expression in advanced-stage Ewing sarcoma: implications for immune recognition. J Pathol 218: 222–231. 19274709 10.1002/path.2537

[bib18] Nemunaitis, J (2011). Multifunctional vaccines in cancer: the ‘triad' approach. Expert Rev Vaccines 10: 713–715. 21692693 10.1586/erv.11.78

[bib19] Senzer, N, Barve, M, Kuhn, J, Melnyk, A, Beitsch, P, Lazar, M et al. (2012). Phase I trial of “bi-shRNAi(furin)/GMCSF DNA/autologous tumor cell” vaccine (FANG) in advanced cancer. Mol Ther 20: 679–686. 22186789 10.1038/mt.2011.269 PMC3293620

[bib20] Senzer, N, Barve, M, Nemunaitis, J, Kuhn, J, Melnyk, A, Beitsch, P et al. (2013). Long term follow up: phase I trial of “bi-shRNA furin/GMCSF DNA/autologous tumor cell” immunotherapy (FANG™) in advanced cancer. J Vaccines Vaccin 4: 209.

[bib21] Maples, PB, Kumar, P, Yu, Y, Wang, Z, Jay, C, Pappen, BO et al. (2010). FANG vaccine: autologous tumor vaccine genetically modified to express GM-CSF and block production of furin. BioProcess J 8: 4–14.

[bib22] Olivares, J, Kumar, P, Yu, Y, Maples, PB, Senzer, N, Bedell, C et al. (2011). Phase I trial of TGF-beta 2 antisense GM-CSF gene-modified autologous tumor cell (TAG) vaccine. Clin Cancer Res 17: 183–192. 21208907 10.1158/1078-0432.CCR-10-2195

[bib23] Thiel, U, Pirson, S, Müller-Spahn, C, Conrad, H, Busch, DH, Bernhard, H et al. (2011). Specific recognition and inhibition of Ewing tumour growth by antigen-specific allo-restricted cytotoxic T cells. Br J Cancer 104: 948–956. 21407224 10.1038/bjc.2011.54 PMC3065285

[bib24] Mahlendorf, DE and Staege, MS (2013). Characterization of Ewing sarcoma associated cancer/testis antigens. Cancer Biol Ther 14: 254–261. 23291981 10.4161/cbt.23298 PMC3595308

[bib25] Yabe, H, Tsukahara, T, Kawaguchi, S, Wada, T, Torigoe, T, Sato, N et al. (2011). Prognostic significance of HLA class I expression in Ewing's sarcoma family of tumors. J Surg Oncol 103: 380–385. 21400519 10.1002/jso.21829

[bib26] Peters, HL, Yan, Y, Nordgren, TM, Cutucache, CE, Joshi, SS and Solheim, JC (2013). Amyloid precursor-like protein 2 suppresses irradiation-induced apoptosis in Ewing sarcoma cells and is elevated in immune-evasive Ewing sarcoma cells. Cancer Biol Ther 14: 752–760. 23792571 10.4161/cbt.25183 PMC3841215

[bib27] Kailayangiri, S, Altvater, B, Meltzer, J, Pscherer, S, Luecke, A, Dierkes, C et al. (2012). The ganglioside antigen G(D2) is surface-expressed in Ewing sarcoma and allows for MHC-independent immune targeting. Br J Cancer 106: 1123–1133. 22374462 10.1038/bjc.2012.57 PMC3304425

[bib28] Liebsch, L, Kailayangiri, S, Beck, L, Altvater, B, Koch, R, Dierkes, C et al. (2013). Ewing sarcoma dissemination and response to T-cell therapy in mice assessed by whole-body magnetic resonance imaging. Br J Cancer 109: 658–666. 23839490 10.1038/bjc.2013.356 PMC3738111

[bib29] Cho, D, Shook, DR, Shimasaki, N, Chang, YH, Fujisaki, H and Campana, D (2010). Cytotoxicity of activated natural killer cells against pediatric solid tumors. Clin Cancer Res 16: 3901–3909. 20542985 10.1158/1078-0432.CCR-10-0735 PMC3168562

[bib30] Mackall, CL, Rhee, EH, Read, EJ, Khuu, HM, Leitman, SF, Bernstein, D et al. (2008). A pilot study of consolidative immunotherapy in patients with high-risk pediatric sarcomas. Clin Cancer Res 14: 4850–4858. 18676758 10.1158/1078-0432.CCR-07-4065 PMC2497450

[bib31] Sporn, MB, Roberts, AB, Wakefield, LM and Assoian, RK (1986). Transforming growth factor-beta: biological function and chemical structure. Science 233: 532–534. 3487831 10.1126/science.3487831

[bib32] Massagué, J (1987). The TGF-beta family of growth and differentiation factors. Cell 49: 437–438. 3471351 10.1016/0092-8674(87)90443-0

[bib33] Border, WA and Ruoslahti, E (1992). Transforming growth factor-beta in disease: the dark side of tissue repair. J Clin Invest 90: 1–7. 1634602 10.1172/JCI115821 PMC443055

[bib34] Jachimczak, P, Bogdahn, U, Schneider, J, Behl, C, Meixensberger, J, Apfel, R et al. (1993). The effect of transforming growth factor-beta 2-specific phosphorothioate-anti-sense oligodeoxynucleotides in reversing cellular immunosuppression in malignant glioma. J Neurosurg 78: 944–951. 8487077 10.3171/jns.1993.78.6.0944

[bib35] Merzak, A, McCrea, S, Koocheckpour, S and Pilkington, GJ (1994). Control of human glioma cell growth, migration and invasion *in vitro* by transforming growth factor beta 1. Br J Cancer 70: 199–203. 8054266 10.1038/bjc.1994.280 PMC2033507

[bib36] Jennings, MT, Kaariainen, IT, Gold, L, Maciunas, RJ and Commers, PA (1994). TGF beta 1 and TGF beta 2 are potential growth regulators for medulloblastomas, primitive neuroectodermal tumors, and ependymomas: evidence in support of an autocrine hypothesis. Hum Pathol 25: 464–475. 8200640 10.1016/0046-8177(94)90118-x

[bib37] Jennings, MT and Pietenpol, JA (1998). The role of transforming growth factor beta in glioma progression. J Neurooncol 36: 123–140. 9525812 10.1023/a:1005863419880

[bib38] Ashley, DM, Kong, FM, Bigner, DD and Hale, LP (1998). Endogenous expression of transforming growth factor beta1 inhibits growth and tumorigenicity and enhances Fas-mediated apoptosis in a murine high-grade glioma model. Cancer Res 58: 302–309. 9443409

[bib39] Ashley, DM, Sampson, JH, Archer, GE, Hale, LP and Bigner, DD (1998). Local production of TGF beta1 inhibits cerebral edema, enhances TNF-alpha induced apoptosis and improves survival in a murine glioma model. J Neuroimmunol 86: 46–52. 9655471 10.1016/s0165-5728(98)00017-4

[bib40] Bierie, B and Moses, HL (2006). Tumour microenvironment: TGFbeta: the molecular Jekyll and Hyde of cancer. Nat Rev Cancer 6: 506–520. 16794634 10.1038/nrc1926

[bib41] Pardali, K and Moustakas, A (2007). Actions of TGF-beta as tumor suppressor and pro-metastatic factor in human cancer. Biochim Biophys Acta 1775: 21–62. 16904831 10.1016/j.bbcan.2006.06.004

[bib42] Hanahan, D and Weinberg, RA (2000). The hallmarks of cancer. Cell 100: 57–70. 10647931 10.1016/s0092-8674(00)81683-9

[bib43] Levy, L and Hill, CS (2006). Alterations in components of the TGF-beta superfamily signaling pathways in human cancer. Cytokine Growth Factor Rev 17: 41–58. 16310402 10.1016/j.cytogfr.2005.09.009

[bib44] Sorbye, SW, Kilvaer, TK, Valkov, A, Donnem, T, Smeland, E, Al-Shibli, K et al. (2012). Prognostic impact of CD57, CD68, M-CSF, CSF-1R, Ki67 and TGF-beta in soft tissue sarcomas. BMC Clin Pathol 12: 7. 22554285 10.1186/1472-6890-12-7 PMC3408340

[bib45] Bodmer, S, Strommer, K, Frei, K, Siepl, C, de Tribolet, N, Heid, I et al. (1989). Immunosuppression and transforming growth factor-beta in glioblastoma. Preferential production of transforming growth factor-beta 2. J Immunol 143: 3222–3229. 2809198

[bib46] Chen, TC, Hinton, DR, Yong, VW and Hofman, FM (1997). TGF-B2 and soluble p55 TNFR modulate VCAM-1 expression in glioma cells and brain derived endothelial cells. J Neuroimmunol 73: 155–161. 9058771 10.1016/s0165-5728(96)00190-7

[bib47] Li, MO, Wan, YY, Sanjabi, S, Robertson, AK and Flavell, RA (2006). Transforming growth factor-beta regulation of immune responses. Annu Rev Immunol 24: 99–146. 16551245 10.1146/annurev.immunol.24.021605.090737

[bib48] Fakhrai, H, Dorigo, O, Shawler, DL, Lin, H, Mercola, D, Black, KL et al. (1996). Eradication of established intracranial rat gliomas by transforming growth factor beta antisense gene therapy. Proc Natl Acad Sci USA 93: 2909–2914. 8610141 10.1073/pnas.93.7.2909 PMC39733

[bib49] Scarlett, UK, Rutkowski, MR, Rauwerdink, AM, Fields, J, Escovar-Fadul, X, Baird, J et al. (2012). Ovarian cancer progression is controlled by phenotypic changes in dendritic cells. J Exp Med 209: 495–506. 22351930 10.1084/jem.20111413 PMC3302234

[bib50] Thomas, DA and Massagué, J (2005). TGF-beta directly targets cytotoxic T cell functions during tumor evasion of immune surveillance. Cancer Cell 8: 369–380. 16286245 10.1016/j.ccr.2005.10.012

[bib51] Rook, AH, Kehrl, JH, Wakefield, LM, Roberts, AB, Sporn, MB, Burlington, DB et al. (1986). Effects of transforming growth factor beta on the functions of natural killer cells: depressed cytolytic activity and blunting of interferon responsiveness. J Immunol 136: 3916–3920. 2871107

[bib52] Kasid, A, Bell, GI and Director, EP (1988). Effects of transforming growth factor-beta on human lymphokine-activated killer cell precursors. Autocrine inhibition of cellular proliferation and differentiation to immune killer cells. J Immunol 141: 690–698. 3133414

[bib53] Tsunawaki, S, Sporn, M, Ding, A and Nathan, C (1988). Deactivation of macrophages by transforming growth factor-beta. Nature 334: 260–262. 3041283 10.1038/334260a0

[bib54] Hirte, H and Clark, DA (1991). Generation of lymphokine-activated killer cells in human ovarian carcinoma ascitic fluid: identification of transforming growth factor-beta as a suppressive factor. Cancer Immunol Immunother 32: 296–302. 1998971 10.1007/BF01789047 PMC11038957

[bib55] Ruffini, PA, Rivoltini, L, Silvani, A, Boiardi, A and Parmiani, G (1993). Factors, including transforming growth factor beta, released in the glioblastoma residual cavity, impair activity of adherent lymphokine-activated killer cells. Cancer Immunol Immunother 36: 409–416. 8500113 10.1007/BF01742258 PMC11038209

[bib56] Naganuma, H, Sasaki, A, Satoh, E, Nagasaka, M, Nakano, S, Isoe, S et al. (1996). Transforming growth factor-beta inhibits interferon-gamma secretion by lymphokine-activated killer cells stimulated with tumor cells. Neurol Med Chir (Tokyo) 36: 789–795. 9420430 10.2176/nmc.36.789

[bib57] Yamaguchi, Y, Tsumura, H, Miwa, M and Inaba, K (1997). Contrasting effects of TGF-beta 1 and TNF-alpha on the development of dendritic cells from progenitors in mouse bone marrow. Stem Cells 15: 144–153. 9090791 10.1002/stem.150144

[bib58] Geissmann, F, Revy, P, Regnault, A, Lepelletier, Y, Dy, M, Brousse, N et al. (1999). TGF-beta 1 prevents the noncognate maturation of human dendritic Langerhans cells. J Immunol 162: 4567–4575. 10201996

[bib59] Steinman, RM, Hawiger, D, Liu, K, Bonifaz, L, Bonnyay, D, Mahnke, K et al. (2003). Dendritic cell function *in vivo* during the steady state: a role in peripheral tolerance. Ann NY Acad Sci 987: 15–25. 12727620 10.1111/j.1749-6632.2003.tb06029.x

[bib60] Ashcroft, GS (1999). Bidirectional regulation of macrophage function by TGF-beta. Microbes Infect 1: 1275–1282. 10611755 10.1016/s1286-4579(99)00257-9

[bib61] Takeuchi, M, Alard, P and Streilein, JW (1998). TGF-beta promotes immune deviation by altering accessory signals of antigen-presenting cells. J Immunol 160: 1589–1597. 9469414

[bib62] Du, C and Sriram, S (1998). Mechanism of inhibition of LPS-induced IL-12p40 production by IL-10 and TGF-beta in ANA-1 cells. J Leukoc Biol 64: 92–97. 9665281 10.1002/jlb.64.1.92

[bib63] Nemunaitis, JJ, Senzer, NN, Barve, MA, Oh, J, Kumar, P, Rao, D et al. (2014). Survival effect of bi-shRNAfurin/GMCSF DNA based immunotherapy (FANG™) in 123 advanced cancer patients to alpha-interferon-ELISPOT response. J Clin Oncol 32: 5s (suppl; abstr 3077).

[bib64] Oh, J, Barve, M, Bedell, C, Kuhn, J, Fine, B, Heffernan, TP et al. (2014). Randomized Phase II Trial of Adjuvant Autologous Tumor Cell Vaccine (FANG™) for High Risk Stage III/IV Ovarian Cancer: Preliminary Results (Poster #624). In ASGCT 17th Annual Meeting, Washington, DC.

