# Supplementary material for: Pilot Trial of FANG Immunotherapy in Ewing's Sarcoma
Source: Mol Ther. 2015 Apr 28;23(6):1103–9. doi: 10.1038/mt.2015.43 (PMC4817748; doi:10.1038/mt.2015.43)
Supplement: Supplementary Table S1 — Release criteria of vaccines constructed for treated patients [file mt201543x1.pdf]

SUPPLEMENTARY DATA

Table S1. Release Criteria of Vaccines Constructed for Treated Patients

**Patient #**	**Vaccine ID**	**Vaccine Vials**	**Average Cell Viability (%)**	**GMCSF Expression (pg)**	**%TGFb1 Knockdown**	**%TGFb2 Knockdown**
1	058	10	97	31	88%	84%
2	062*	8	84	5160	100%	99%
	098*	6	89	500	100%	99%
3	063	12	*97*	1975	95%	96%
4	081	4	*97*	3794	98%	89%
5	083	4	*93*	184	100%	100%
6	089	8	91	2084	100%	98%
7	090	12	95	1387	100%	99%
8	092	9	89	14751	100%	100%
9	095	6	93	1975	96%	100%
10	101	7	88	1906	92	NE
11	104	4	91	678	99%	100%
12	107	12	93	1810	100%	95%

* 062 & 098 are sequential immunotherapies constructed for 1 patient.

NE = Not Evaluable
